# Endogenous melatonin promotes rhythmic recruitment of neutrophils toward an injury in zebrafish

**DOI:** 10.1038/s41598-017-05074-w

**Published:** 2017-07-05

**Authors:** Da-long Ren, Cheng Ji, Xiao-Bo Wang, Han Wang, Bing Hu

**Affiliations:** 1 Chinese Academy of Sciences Key Laboratory of Brain Function and Disease, School of Life Sciences, University of Science and Technology of China, No. 96 Jinzhai Road, Hefei, Anhui Province 230026 P. R. China; 2 Center for Circadian Clocks, Soochow University, Suzhou, 215123 Jiangsu China; 3 School of Biology & Basic Medical Sciences, Medical College, Soochow University, Suzhou, 215123 Jiangsu China

## Abstract

Neutrophil recruitment to injured tissue appears to be an evolutionarily conserved strategy for organisms to fight against exogenous insults. Recent studies have shown rhythmic migration of neutrophils and several factors, including melatonin, have been implicated in regulating this rhythmic migration. The mechanisms underlying how endogenous melatonin regulates rhythmic neutrophils migration, however, are unclear. Here we generated a zebrafish *annat2* mutant that lacks endogenous melatonin and, subsequently, a *Tg*(*lyz:EGFP*)*;aanat2* ^−/−^ transgenic line that allows for monitoring neutrophils migration visually in live zebrafish. We observed that migrating neutrophils are significantly reduced in *aanat2* ^−/−^ mutant zebrafish under a light/dark condition, and the disrupted migrating rhythmicity of neutrophils in *aanat2* ^−/−^ zebrafish is independent of the circadian clock. Further, we also found that endogenous melatonin enhances neutrophils migration likely by inducing the expression of cytokines such as interleukin-8 and interleukin-1β. Together, our findings provide evidence that endogenous melatonin promotes rhythmic migration of neutrophils through cytokines in zebrafish.

## Introduction

Approximately half of circulating white blood cells in mammals are neutrophils, which are the first cellular defenders against exogenous insults executed by the innate immune system^1–3^. An understanding of neutrophilic inflammation would undoubtedly provide an important approach to the development of treatments for tissue damage caused by aberrant neutrophil recruitment. Many lines of evidence have revealed invaluable insights into traditional neutrophils migration^4–7^. However, little is known about circadian roles in the immune system, especially rhythmic migration of neutrophils.

A growing body of studies has demonstrated that the circadian clock plays a regulatory role in various immune processes^8^. Circadian clock genes were shown to exhibit oscillating expression in peripheral blood mononuclear cells and NK cells^9, 10^, and the immune cells display marked rhythmicity in TNF-α and IL-6 secretion stimulated by endotoxins at different circadian times^11^. In addition, time-of-day variation in the sensitivity and vulnerability to infection^12, 13^ as well as occurrences of inflammation-related diseases, such as rheumatoid arthritis and myocardial infarction^14–16^, all implicate circadian roles in immunological activities. Previous reports further showed that neutrophil recruitment to the injury also exhibits circadian rhythmicity in zebrafish, which could be regulated by melatonin^17^. However, lack of melatonin-deficient zebrafish mutant lines has prevented examining the roles of endogenous melatonin in the regulation of rhythmic neutrophils migration.

Melatonin synthesis exhibits robust rhythmicity as the gene arylalkylamine N-acetyltransferase 2 (*aanat2*) encoding its rate-limiting enzyme is tightly controlled by the circadian clock^18^. Melatonin has been shown to mediate numerous circadian output processes^19, 20^ such as the sleep-wake cycle and immune processes^21–24^. In particular, the role of melatonin in regulating inflammation is controversial^24^. Exogenous melatonin was reported to cause anti-inflammatory effects in various animals^22, 25^. In contrast, inflammatory effects were not observed in pinealectomized rodents whose endogenous melatonin is dramatically depleted^26–28^. To address these inconsistent effects of melatonin, we set out to investigate how endogenous melatonin regulates neutrophil recruitment and its rhythmicity in zebrafish.

Zebrafish have emerged as a powerful model for studying innate immune functions, as their embryos are transparent, allowing for real-time visualization of fluorescent proteins at the single-cell level *in vivo* ^3^. As a diurnal animal, zebrafish have been attractive for studying the circadian rhythm because of their conserved clock mechanisms shared with mammals^29^. In this study, we generated an *annat2* mutant zebrafish line with CRISPR-Cas9, and also the *Tg*(*lyz:EGFP*)*;aanat2* ^−/−^ zebrafish line. Through visualizing rhythmic migration of neutrophils *in vivo*, we revealed that endogenous melatonin regulates this rhythmic neutrophils migration. Although normal oscillations of circadian clock genes are disrupted in *aanat2* ^−/−^ fish, reduced neutrophils migration still persists in *aanat2* ^−/−^ larvae. We also found that endogenous melatonin enhances neutrophils migration likely through inducing the expression of cytokines. These results demonstrated that endogenous melatonin participates in the regulation of neutrophil rhythmic migration in zebrafish.

## Materials and Methods

### Zebrafish lines and maintenance

Zebrafish embryos were harvested from natural matings of wild-type (AB), transgenic *Tg*(*lyz:EGFP*) labeled with neutrophils and *aanat2* ^−/−^ lines. The *Tg*(*lyz:EGFP*)*;aanat2* ^−/−^ line was obtained by crossing *aanat2* ^−/−^ and Tg(*lyz*:EGFP) for two consecutive generations. Embryos were maintained in 14/10 light/dark (LD) conditions at 28.5 °C. N-phenylthiourea (PTU, Sigma, USA) was used to prevent pigment formation. All animal manipulations were conducted in strict accordance with the guidelines and regulations set forth by the University of Science and Technology of China (USTC) Animal Resources Center and University Animal Care and Use Committee. The protocol was approved by the Committee on the Ethics of Animal Experiments of the USTC (Permit Number: USTCACUC1103013). All zebrafish surgeries were performed after anesthetization with Tricaine methane-sulfonate (MS-222, Sigma) treatment.

### Design of the CRISPR-Cas9-targeted site and synthesis of Cas9 and gRNA

The gRNA was designed to target the first exon of zebrafish *aanat2* by “seqbuilder” software (DNAStar, USA) according to the 5′-GGNNNNNNNNNNNNNNNNNNNG G-3′ form^30^. The targetingsequence started with GG, ended with NGG (PAM) and also contained a restrictive enzyme MspI near the PAM for genotyping. The Cas9 mRNA and gRNA were synthesized as described previously with modification^31^. Briefly, the Cas9 mRNA was synthesized using a T7 mMESSAGE mMACHINE Kit (Ambion, USA). The DNA fragment of the gRNA was amplified by PCR with a pair of primers (Table 1), and then purified by phenol and chloroform. The gRNA was *in vitro* transcribed with SP6 Riboprobe Systems (Promega, USA).

**Table 1:** Primers used in the experiment.

Gene	Note	Forward primer(5′-3′)	Reverse primer (5′-3′)
*aanat2*	gRNA	GATCACTAATACGACTCACTATAGGCAAAGACGACACACGTTACGTTTTAGAGCTAGAAAT	AAAAGCACCGACTCGGTGCC
*aanat2*	CRISPR-Cas9	CTAAAGTGTGCGCGTGTCAG	AGAACTACTGGCACTTTGAGACA
*clock1a*	qRT-PCR	AGCAGGGACAGAACCAGG	GTGTTGCGGTTGTGAATG
*bmal1a*	qRT-PCR	GAAGACATTACGAGGGGCCA	AGAGGAAACCATCAGCAGCC
*bmal1b*	qRT-PCR	CCCTCTAGCTGTGGCTCAAG	TCCCGCCATTGGACATCTTT
*per1b*	qRT-PCR	AGGAAGGCTGACAGATGATGAATG	CCAGAGTGGGCTAAAGCGAAGTA
*per2*	qRT-PCR	ACGAGGACAAGCCAGAGGAACG	GCACTGGCTGGTGATGGAGA
*cry1bb*	qRT-PCR	TCTACCAACAACTGTCCCGCTAC	GCCATCCCATTTCCATTCCC
*tnf-α*	qRT-PCR	GCGCTTTTCTGAATCCTACG	TGCCCAGTCTGTCTCCTTCT
*il-1β*	qRT-PCR	GTACTCAAGGAGATCAGCGG	CTCGGTGTCTTTCCTGTCCA
*il-8*	qRT-PCR	CCACACACACTCCACACACA	CCACTGAATTGTCCTTTCATCA
*il-6*	qRT-PCR	GCTATTCCTGTCTGCTACACTGG	TGAGGAGAGGAGTGCTGATCC
*β-actin*	qRT-PCR	ACGAACGACCAACCTAAACTCT	TTAGACAACTACCTCCCTTTGC
*aanat2*	cDNA	CGCGGATCCATGATGGCACCGCAGGTCGTCA	CCGGAATTCCTAACATCCGCTGTTTCGTCGTGC
*aanat2*	anti-cDNA	CCGGAATTCATGATGGCACCGCAGGTCGTCA	CGCGGATCCCTAACATCCGCTGTTTCGTCGTGC

### Analysis of mutagenesis frequencies and identification of *aanat2* mutants

Cas9 mRNA and *aanat2* gRNA were co-microinjected into one-cell zebrafish embryos. Genomic DNAs of three groups (15 embryos each), including wild-type controls, were extracted at 24 hours post-fertilization (hpf), and used as templates for PCR. A 290-bp DNA fragment containing the *aanat2* target fragment was PCR amplified, digested with MspI (New England Biolabs, UK) at 37 °C for 3 h, and electrophoresed on a 3% agarose gel. Intensities of cleaved and uncleaved bands were quantified with Image J software (NIH, USA). The uncleaved bands were recovered after gel electrophoresis and cloned into pMD-19T (Takara, Japan), and single clones were picked up for PCR and restriction enzyme digestion, and then sequenced by Sanger sequencing (GENEWIZ, Inc.). Primers used in the experiment are listed in Table 1.

The microinjected founder (F_0_) embryos were raised to adulthood and then crossed with wild-type zebrafish to produce F_1_ embryos. From each cross, 15 F_1_ embryos were collected for genomic DNA extraction and enzymatic digestion. The F_1_ embryos that carry heritable mutations were raised to adulthood, and then each individual F_1_ fish was identified with PCR amplication of fin-clipped DNAs and enzymatic digestion. Homozygous *aanat2* mutant fish were generated by crossing of the male and female fish carrying the same mutation.

### Tail fin injury and live imaging

Zebrafish larval tail fin was transected at the end of the spinal cord by a sterile blade on a plastic petri dish after being anesthetized with Tricaine methane-sulfonate^22^ (Sigma, USA). The injured larvae were recovered at 28.5 °C in embryo medium until live imaging. Three hours after wounding, transgenic larvae *Tg*(*lyz:EGFP*) labeled with neutrophils as well as *Tg*(*lyz:EGFP*)*;aanat2* ^−/−^ were embedded into low-melting agarose for visual monitoring under an Olympus microscope with a green fluorescent channel.

### Melatonin measurement by ELISA

Melatonin concentrations were detected with an ELISA kit^18^ (IBL international, Germany). Wild-type and *aanat2* mutant larvae were raised under 14/10 LD for 5 days. At 12:00 and 24:00, fifty larvae were collected for ELISA evaluation. Melatonin samples were extracted with a column with methanol according to the kit instructions. The standard curve was generated with a series of concentrations of melatonin. Melatonin content was determined by measuring the optical density with a photometer at 405 nm within 60 min after pipetting of the stop solution.

### RNA extraction and qRT-PCR

Total RNAs were extracted from larvae of wild-type (n = 50) and *aanat2* ^−/−^ (n = 50) at 4-h intervals under LD and DD (dark-dark) conditions using Trizol (Takara, Japan) reagent. Larvae from the cloacal orifice to the incision end were also collected for RNA extraction to examine cytokine expression. Quantitative real-time PCR (qRT-PCR) was conducted with the SYBR green (Invitrogen, USA) system. The clock and cytokine genes were amplified using the profiles of 95 °C, 10 s, 60 °C, 30 s for 40 cycles. qRT-PCR was performed in triplicate with three individual biological samples (nine replicates) at corresponding time points, and the results were normalized to the expression level of the housekeeping gene β-actin and shown as a relative expression level calculated using the2^−ΔΔCt^ method^32^. *P* values were analyzed with one-way analysis of variance (ANOVA) test or Student’s t test.

### Rescue of melatonin content, neutrophils migration and clock gene expression by capped wild-type *aanat2* mRNAs

The zebrafish wild type cDNA and anti-sense cDNA of *aanat2* were cloned into the pCS2+ plasmid with the restriction enzyme BamHI and EcoRI, and linearized with SacII. Capped *aanat2* mRNAs and anti-sense mRNAs were transcribed from the linearized plasmids using the mMACHINE *in-vitro* transcription kit (SP6; Ambion, Austin, TX, USA) according to the manufacturer’s instructions. To be specific, 2 μl 10X reaction buffer, 2 μl enzyme mix, 10 μl 2X NTP/CAP, 1 μl RNase Inhibitor and 1 μg plasmid DNA were mixed, and nuclease-free water was added to the mix solution up to 20 μl and incubate at 37 °C for 2 hr. mRNAs identification were using DNA gel electrophoresis and the content of mRNAs were measured using the spectrophotometer (Nanodrop2000, Thermo). 200 ng/μl *aanat2* capped mRNAs or 200 ng/μl anti-sense capped mRNAs were microinjected into one-cell of zebrafish wild type, *aanat2* ^−/−^ embryos or *Tg*(*lyz:EGFP*)*;aanat2* ^−/−^ embryos. None microinjected embryos of wild type, *aanat2* ^−/−^ or *Tg*(*lyz:EGFP*)*;aanat2* ^−/−^ were as controls. Total RNAs were extracted from 50 larvae of ZT12 each sample.

### Statistical analysis

All experiments were independently repeated three times. The data were analyzed with an unpaired, two-tailed *t*-test, one-way ANOVA using GraphPad Prism version 5.0 (Prism, USA). The results are shown as the mean ± SEM. The level of significance was set to *P* < 0.05. *, **, and *** represent *P* < 0.05, *P* < 0.01, and *P* < 0.001, respectively.

## Results

### Generation of *aanat2*^−/−^ zebrafish using CRISPR-Cas9

**Figure 1:**
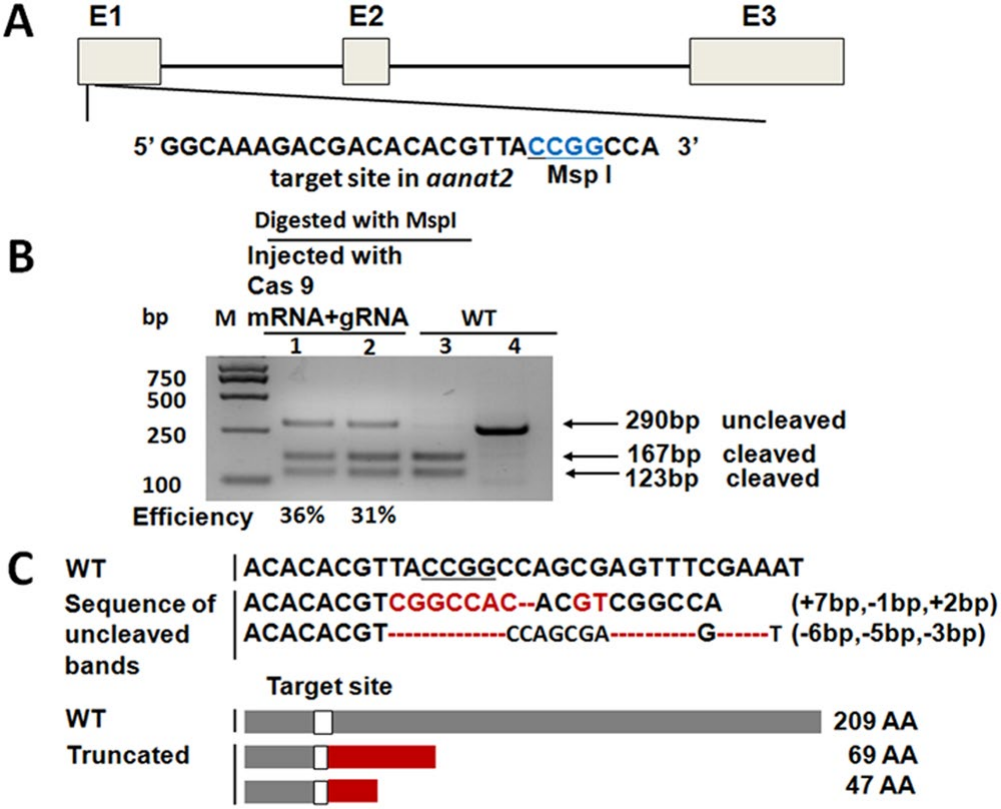
Generation of *aanat2* mutant zebrafish using CRISPR-Cas9. (**A**) Schematic of the Cas9-gRNA-targeted site in the first *aanat2* exon. The protospacer-adjacent motif (PAM) sequence (CGG) is labeled in blue and the MspI restriction site is underlined. (**B**) The targeted fragment was PCR-amplified from pooled genomic DNA of 15 embryos co-microinjected with 300 pg Cas9 mRNA and 100–200 pg gRNA, and then digested with MspI. The uncleaved (290 bp) and cleaved PCR products (167 bp and 123 bp) were indicated. Mutagenesis efficiencies were calculated by the ratios of intensities of uncleaved bands to the sum of cleaved bands using Image J software. M, marker: 1–2, injected groups at concentrations of 100 pg and 200 pg of *aanat2* gRNA, respectively; 3, wild-type control with digestion of MspI; 4, undigested wild-type PCR products. (**C**) Representative sequencing results of the two mutated fish lines. One had a 9-bp insertion and a 1-bp deletion, the other had a 14-bp deletion (upper), and both lines had frameshift mutations that resulted in truncated proteins (lower). AA, amino acids.

Our previous study showed that migrating neutrophils display a robust daily rhythm^17^. To further investigate the role of endogenous melatonin in rhythmic neutrophils migration, we generated *aanat2* mutant lines. Aannat2 is the rate-limiting enzyme for melatonin synthesis in vertebrates^33, 34^. Using “Seqbuilder” software, we designed a CRISPR-Cas9-targeted site in the first exon of zebrafish *aanat2*, which also contains an MspI restriction site for evaluating and screening mutants (Fig. 1A). *In vitro* synthesized capped Cas9 mRNAs and gRNA were then microinjected simultaneously into one-cell embryos. To evaluate the mutant efficiency, a 290-bp targeted DNA fragment was PCR amplified and digested with the MspI restriction enzyme. Results showed that the mutant efficiency was approximately 32–51% in F_0_ larvae (Fig. 1B). Representative sequencing results of the two mutated fish lines showed that one had a 9-bp insertion and 1-bp deletion, the other had a 14-bp deletion, and both lines had frame-shift mutations (Fig. 1C). The mutant lines with the 9-bp insertion and 1-bp deletion were used in the study.

### Establishment of a visual model monitoring neutrophils migration in *aanat2* mutant zebrafish

**Figure 2:**
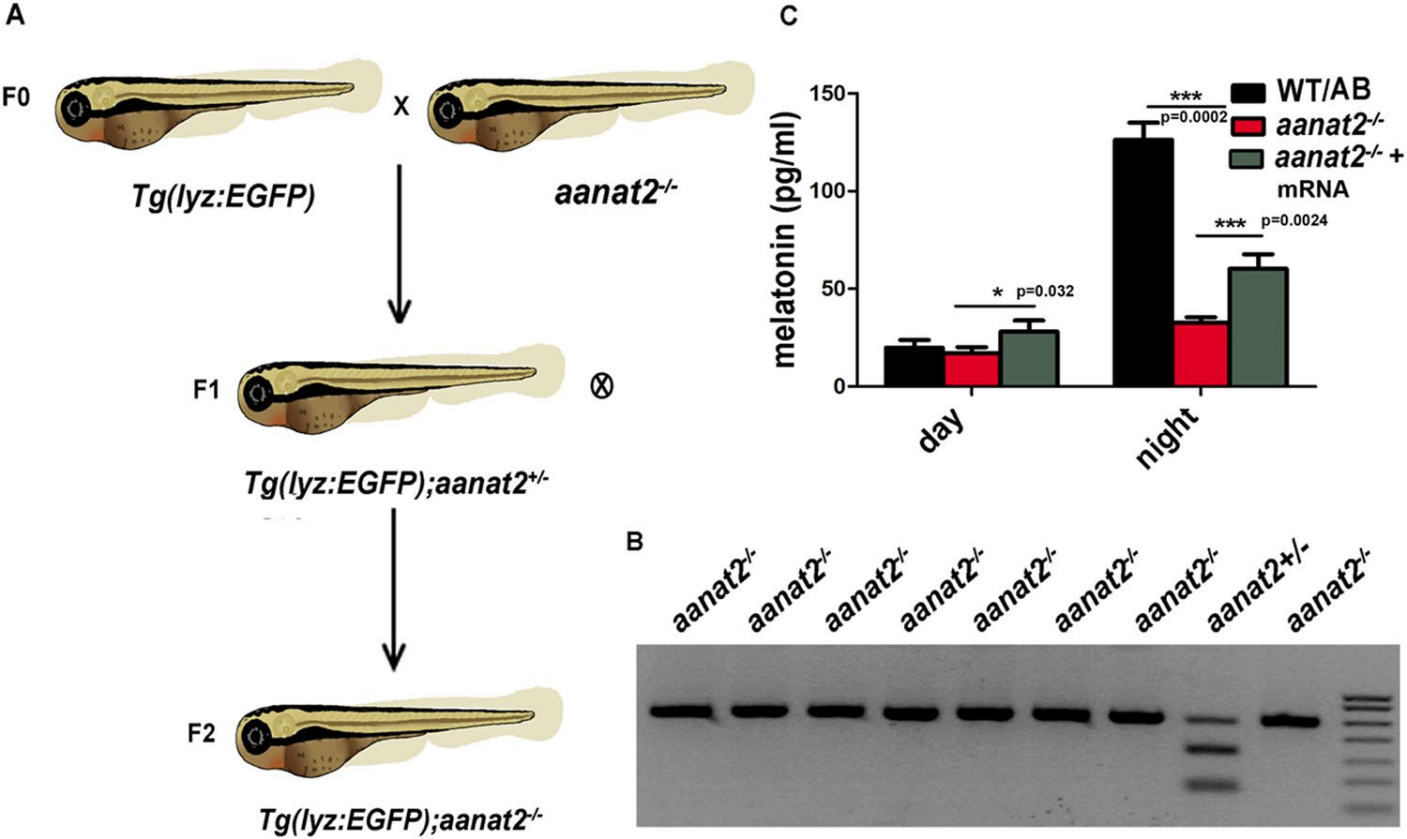
Establishment of a visual model for monitoring neutrophil migration in *aanat2* ^−/−^ zebrafish. (**A**) Hybridization of transgenic line *Tg*(*lyz:EGFP*) labeling neutrophils and *aanat2* mutants for two consecutive generations. (**B**) In F_2_ zebrafish, we identified the homozygous *aanat2* ^−/−^ transgenic zebrafish. Extracted genomic DNA from the tail fin of screened transgenic zebrafish was amplified using PCR and digested using the MspI enzyme. The uncleaved band indicated homozygous zebrafish. (**C**) To evaluate the functional effect of *Tg*(*lyz:EGFP*)*;aanat2* ^−/−^ zebrafish, we measured melatonin levels using an ELISA. Fifty larvae were homogenated and then melatonin was extracted using methanol as an individual sample (IBL international, Germany). The experiment used three samples. Results indicated that melatonin was significantly decreased at night compared with WT/AB larvae. The melatonin content could be partly rescued by injection of *aanat2* capped mRNA (control, n = 50; mutant, n = 50; mutant + mRNA, n = 50) (ANOVA analysis). (***P < 0.001).

To monitor neutrophils migration in live zebrafish in a real-time manner, we crossed transgenic line *Tg*(*lyz:EGFP*) labeling neutrophils with *aanat2* mutant zebrafish (Fig. 2A). Then we screened the homozygous *aanat2* mutant whose neutrophils are also labeled with green fluorescent protein, called *Tg*(*lyz:EGFP*)*;aanat2* ^−/−^ fish, using enzymatic digestion of fin-clipped DNAs (Fig. 2B). We also determined the melatonin content in *Tg*(*lyz:EGFP*)*;aanat2* ^−/−^ larvae with ELISA. Results showed that melatonin is at a very low concentration in *aanat2* ^−/−^ larvae during day and night, while melatonin is dramatically increased in wild-type larvae during the night. The melatonin content could be partly rescued by *aanat2* capped mRNA treatment (Fig. 2C). Moreover, the *aanat2* mutation did not cause a change in zebrafish body weight and length (Supplementary Fig. S1A,B). Hence, neutrophils migration can be visualized in endogenous melatonin-deficient zebrafish.

### Neutrophil recruitment is significantly reduced in *aanat2*^−/−^ larvae

**Figure 3:**
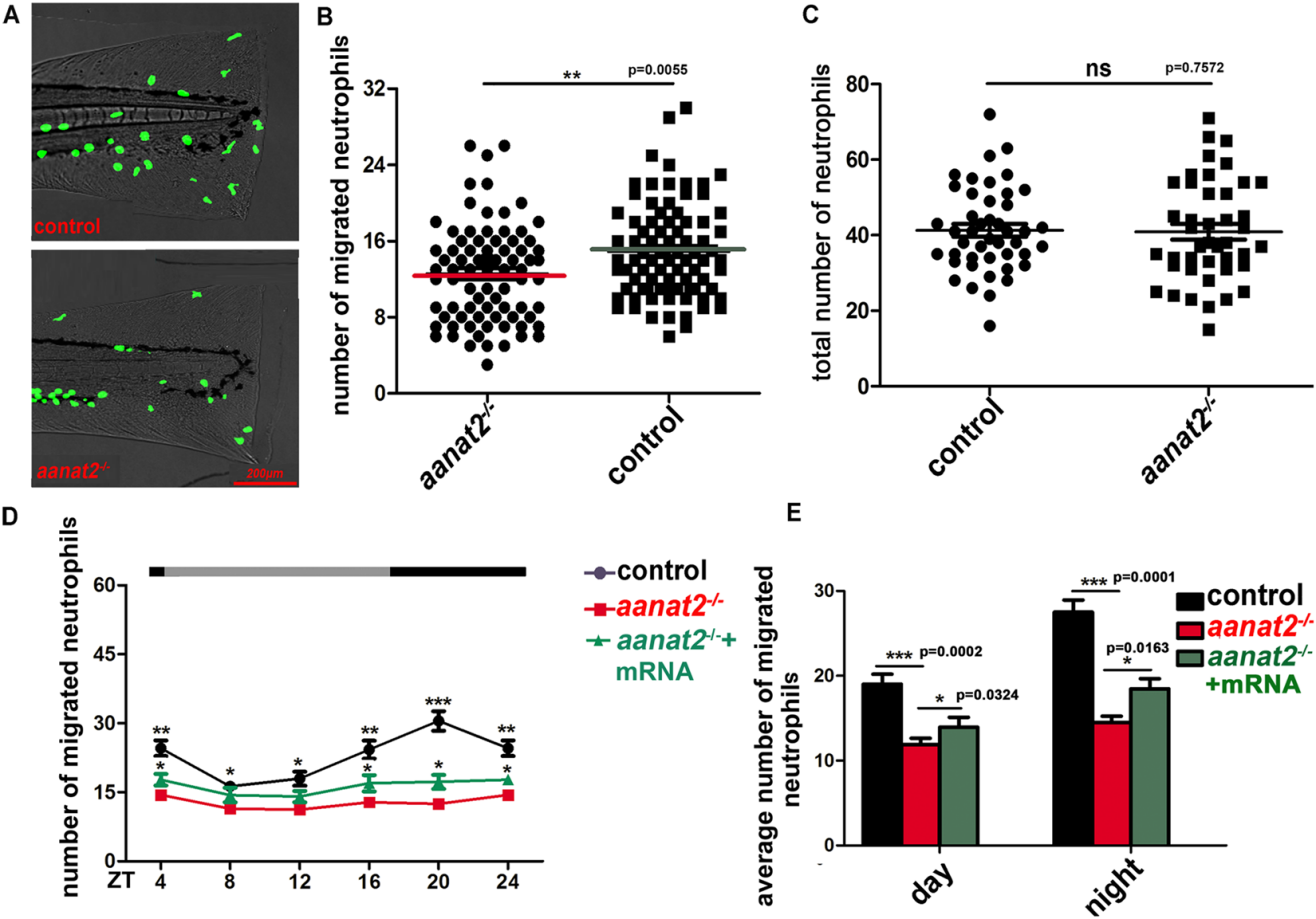
Neutrophil recruitment was significantly reduced in *aanat2* ^−/−^ larvae. (**A**,**B**) Using a tail fin injury model in *Tg*(*lyz:EGFP*)*;aanat2* ^−/−^ zebrafish (4 days), we evaluated the effects of an *aanat2* mutant on neutrophil migration at 12:00 in the day. Results showed that *aanat2* mutants had significantly decreased neutrophil recruitment (control, n = 90; mutant, n = 90). Neutrophils located at 250 μm from the wound ending were counted and regarded as the valid migration number. The experiment was repeated three times (unpaired *t*-test analysis). (**C**) The total number of circulating neutrophils was analyzed by counting fluorescent particles within the 800-μm region from the spinal cord end to the anterior at 12:00. There had no significant difference between the WT/AB (n = 60) and *aanat2* mutant groups (n = 60). The experiment was repeated three times (unpaired *t*-test analysis). Each experiment contains 60 samples. (**D**) To evaluate the rhythmic migration of neutrophils in *aanat2* ^−/−^ zebrafish under LD condition, we monitored neutrophil migration at 4-h intervals in a day-night period using a fluorescent microscope. The results showed that rhythmic recruitment of neutrophils was abolished in *aanat2* ^−/−^ larvae and the migrating neutrophils could be partly rescued by injection of *aanat2* capped mRNA (control, n = 30; mutant, n = 30; mutant + mRNA, n = 30). The data was repeated with three independent experiments (ANOVA analysis). Each experiment contains 30 samples. (**E**) The average number of migrating neutrophils at both day and night was lower in *aanat2* ^−/−^ compared with wild types (control, n = 90; mutant, n = 90). The data was analyzed from Fig. 3D. ZT: zeitgeber times. (**P < 0.01, ***P < 0.001).

Using the injury-induced inflammation model^6, 35^, we examined the effects of endogenous melatonin on rhythmic neutrophil recruitment. Results showed that neutrophil recruitment towards the wound site is significantly reduced in *aanat2* ^−/−^ larvae (Fig. 3A,B). Given the possibility that *aanat2* ^−/−^ may affect the total number of circulating neutrophils, we evaluated the fluorescent neutrophils in *Tg*(*lyz:EGFP*) zebrafish. Considering the difficulty in counting all neutrophils, neutrophils located in the 800-μm region from the spinal cord to the anterior were counted and regarded as the total number, as in a previous study^22^. Results showed that there was no significant difference in the total number of neutrophils between wild-type and *aanat2* ^−/−^ larvae (Fig. 3C). Results also showed that the neutrophil distribution in the control and mutant groups had no significant difference (Supplementary Fig. S1C). We also found that the rhythmic patterns of neutrophils migration were abolished in *aanat2* ^−/−^ mutants (Fig. 3D), consistent with our previous prediction that the rhythmic mode of neutrophils migration is likely disrupted in *aanat2* mutants^17^. The migrating neutrophils could be partly rescued by injection of *aanat2* capped mRNA (Supplementary Fig. S2). Further, the average number of migrating neutrophil was lower in *aanat2* ^−/−^ larvae than in wild types during day and night (Fig. 3E). These results clearly indicated that loss of endogenous melatonin results in reduction of neutrophil recruitment and alters rhythmic neutrophils migration in zebrafish.

### Disrupted expression of circadian clock genes in *aanat2* mutant larvae

**Figure 4:**
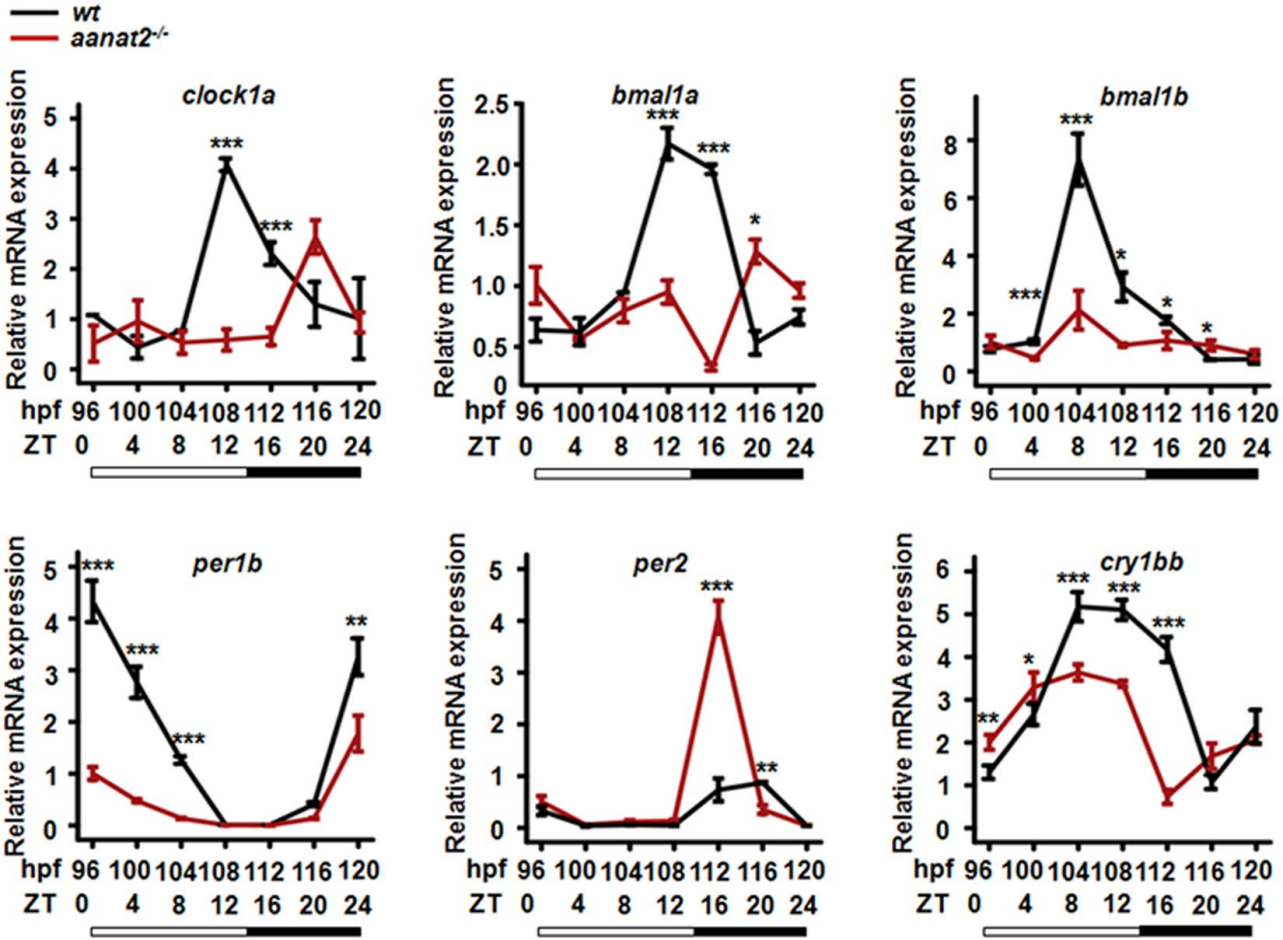
Disrupted expression of circadian clock genes in *aanat2* ^−/−^ fish. Total RNAs were extracted from wild-type and *aanat2* mutant larvae at 4-h intervals for a total of consecutive 24 h under LD. One experiment contains three samples (control = 3, mutant = 3). Each sample contained 50 larvae. The data were analyzed from three samples in both the control and mutant groups. The experiment was repeated three times. (**A**–**F**) qRT-PCR analysis showed that Rhythmic expression patterns of key circadian clock genes, *clock1a*, *bmal1a*, *bmal1b*, *per1a*, *per1b*, *and cry1bb* were all disrupted in *aanat2* mutant larvae. The amplitude of the *per1b* gene was reduced and the oscillation pattern of the *cry1bb* gene was phase shifted. The genes were relative expression to β-actin (ANOVA analysis). (*P < 0.05, **P < 0.01, ***P < 0.001).

Previous studies have shown that exogenous melatonin treatment could alter rhythmic expression of circadian clock genes^36–39^. Here, we examined whether endogenous melatonin deficiency in *aanat2* ^−/−^ could affect the expression of circadian clock genes. qRT-PCR analyses show that *clock1a*, *bmal1a*, *bmal1b and per1b* were significantly down-regulated in *aanat2* ^−/−^ larvae under the LD condition (Fig. 4A–C). While *per2* was up-regulated in *aanat2* ^−/−^ larvae at ZT4 and ZT12.and *cry1bb* exhibited a phase advance of expression in *aanat2* ^−/−^ larvae (Fig. 4E,F). Further, we synthesized wild-type *aanat2* capped mRNAs and microinjected into the mutants to rescue the gene expression at ZT12 as most genes expression was altered at this time point. The rescue assay revealed relative mRNA expression of *clock1a* and *bmal1b* could be rescued by injection of *aanat2* capped mRNA, and relative mRNA expression of *cry1bb* could be partly rescued (Supplementary Fig. S3). Together, these results showed that expression of key circadian clock genes is disrupted in *aanat2* mutant zebrafish.

### Disrupted migrating rhythmicity of neutrophils in *aanat2*^−/−^ zebrafish is independent of the circadian clock

**Figure 5:**
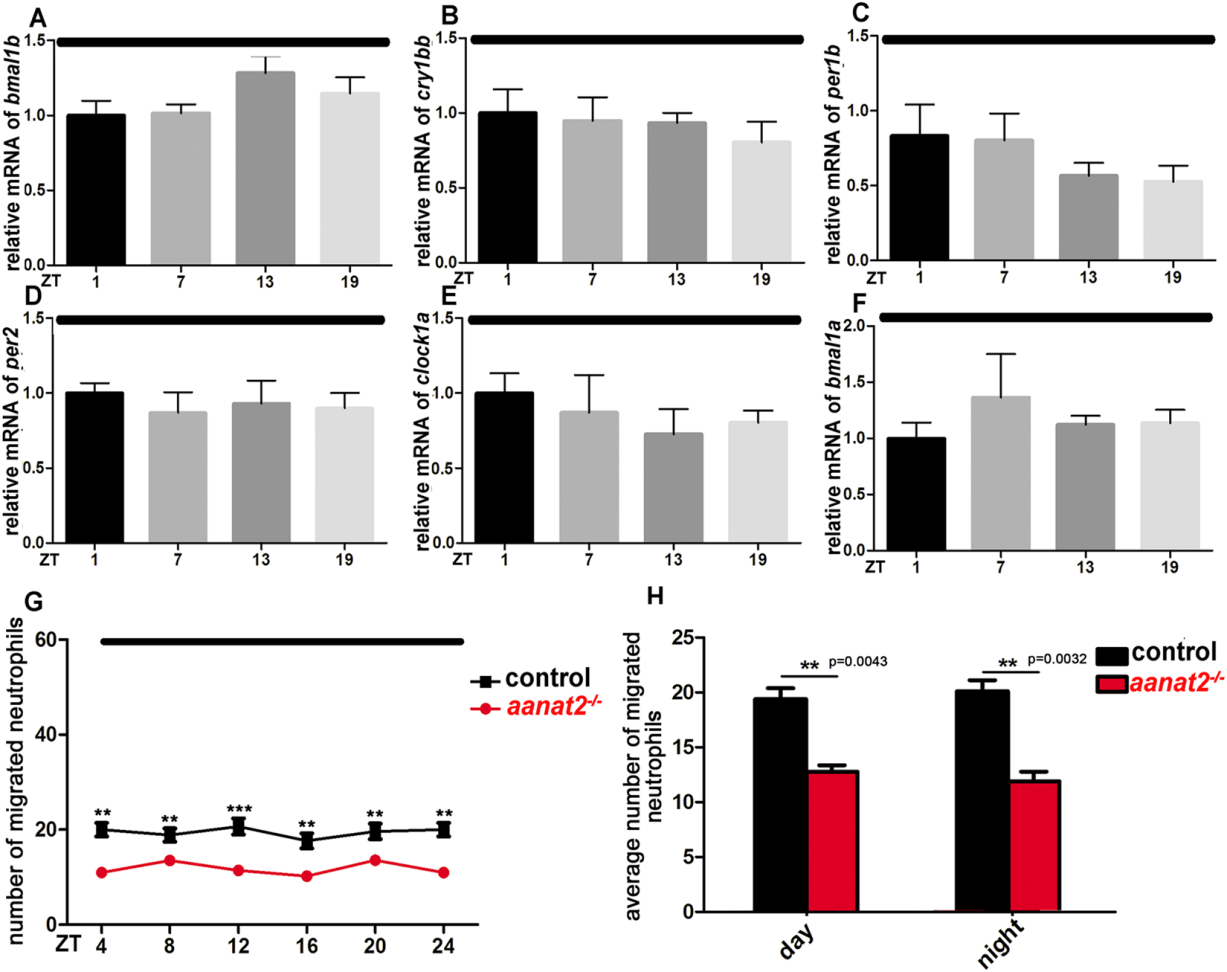
Disrupted migrating rhythmicity of neutrophils in *aanat2* ^−/−^ zebrafish did not require circadian regulation. (**A**–**F**) Embryos were maintained under DD conditions at the onset of fertilization and were examined under the DD condition. qRT-PCR analysis showed that key circadian clock genes *clock1a*, *bmal1a*, *bmal1b*, *per1a*, *per1b*, *and cry1bb* were all expressed at the same level without rhythmicity. Each sample contained 50 embryos. The data were analyzed from three independent samples in both the control and mutant groups. The experiment was repeated three times. (**G**) Neutrophil migration towards the injury is reduced in *aanat2* mutants under the same condition (control, n = 40; mutant, n = 40; mutant + mRNA, n = 40). The experiment was repeated three times. (**H**) The average number of migrating neutrophils was lower in *aanat2* ^−/−^ larvae than wild types during day and night (control, n = 120; mutant, n = 120) (unpaired *t-*test). The data was analyzed from Fig. 5G. The “day” and “night” in constantly-dark are consistent with the time-cycle in nomal light cycle. (**P < 0.01, ***P < 0.001, unpaired *t-*test and ANOVA analysis).

Because of altered expression of key circadian clock genes in *aanat2* ^−/−^ zebrafish, we wondered whether the regulatory role of endogenous melatonin in rhythmic neutrophils migration is a direct effect, or is mediated through a disturbed circadian clock. We raised *aanat2* ^−/−^ embryos at the onset of fertilization under constant darkness (DD), and established arrhythmic zebrafish lacking molecular circadian rhythms^40^. Results showed that oscillating patterns of clock genes *per1b*, *per2*, *cry1bb*, *bmal1a*, *bmal1b* and *clock1a* were abolished and almost all genes were expressed similarly at all circadian time points in these arrhythmic zebrafish larvae (Fig. 5A–F). These results showed that the circadian clock machinery did not function appropriately in these larvae raised continuously from the onset of fertilization under DD. Interestingly, we also observed that, under the same condition, neutrophil recruitment is still significantly reduced in *aanat2* ^−/−^ larvae in a 24-h period (Fig. 5G), and the number of migrating neutrophil averages was lower in *aanat2* ^−/−^ larvae than wild-type controls during day and night under the DD condition (Fig. 5H). Together with our previous study that showed treatment with melatonin at a physiological concentration promotes neutrophils migration in zebrafish^17^, our findings suggest that melatonin promotes neutrophils recruitment rather than through the circadian clock in zebrafish.

### Down-regulation of cytokines in *aanat2*^−/−^ larvae

**Figure 6:**
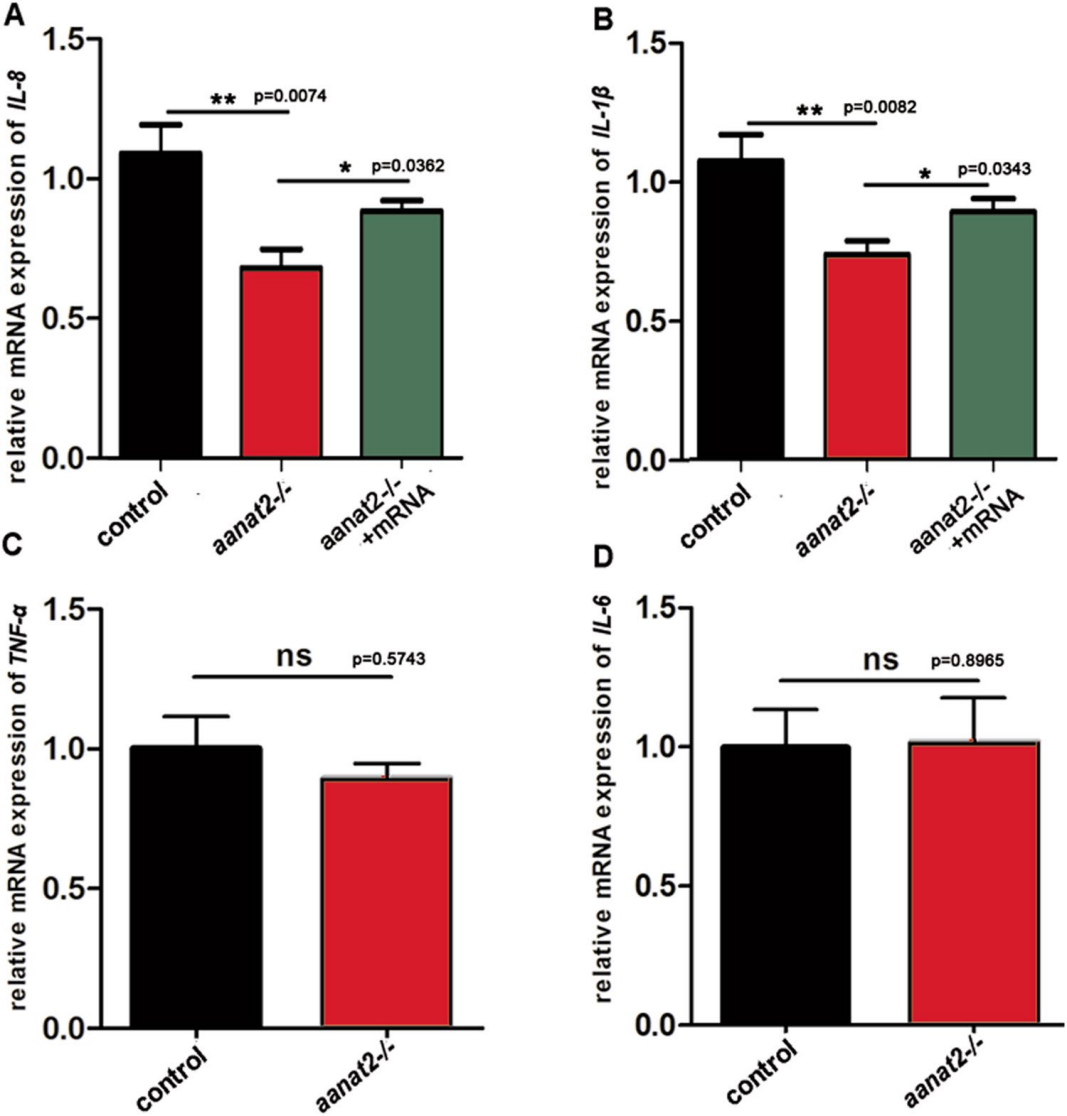
Down-regulation of cytokine expression in *aanat2* ^−/−^ fish. (**A**–**D**) Zebrafish embryos (4 days post fertilization) were collected for RNA extraction at 1.5 h after injury in the darkness. qRT-PCR analysis showed significantly down-regulation of *il-1β* and *il-8*, which could be partly rescued by injection of *aanat2* capped mRNA (one way ANOVA analysis). However, *tnf-α* and *il-6* expressions showed no significant difference between the *aanat2* ^−/−^ and control groups (unpaired *t*-test). Each independent sample contained fifty embryos. The data were analyzed using three samples in both the control and mutant groups. The experiment was repeated three times. (*P < 0.05, **P < 0.01).

Exogenous melatonin also was shown to alter expression of cytokines^24^. We hypothesized that reduced neutrophil recruitment in *aanat2* mutants may be meditated through cytokines. Cytokines were induced using the injury model in darkness as previously described^4^. qRT-PCR results showed that both *il-8* and *il-1β* were significantly down-regulated in *aanat2* mutants (Fig. 6A,B), which could be rescued by *aanat2* capped mRNA injection. The results also showed that *il-8* and *il-1β* exhibited obvious increase in the night period compared with the day period (Supplementary Fig. S4). Both *tnf-α* and *il-6* are barely changed in *aanat2* ^−/−^ larvae (Fig. 6C,D). Similar results also appeared when embryos were raised in DD (data not shown). Previous studies have visually demonstrated that IL-8 directly induces neutrophil recruitment with the eye and optic vesicle model in zebrafish larvae^4, 17^. Taken together, our results implied that endogenous melatonin might promote neutrophils migration by regulating IL-8 expression.

## Discussion

Melatonin has been widely regarded as a regulator of inflammation and circadian rhythms^41–44^. Its role in regulating inflammation and circadian rhythms, however, is controversial^24^, and particularly the function of endogenous melatonin is largely uncertain. Here we generated a melatonin-deficient model in diurnal zebrafish (Fig. 1) and we raised the mutant zebrafish for several generations before conducting all experiments to reduce the off-target effect. *In vivo* imaging of transgenic *lyz:EGFP;aanat2* ^−/−^ larvae using the injury model showed that neutrophils migration is reduced and its migration rhythmicity is abolished in *aanat2* ^−/−^ larvae (Fig. 3). Because rhythmic expression of key circadian clock genes is disrupted in *aanat2* ^−/−^ larvae (Fig. 4), the enhancing effect of melatonin on neutrophils migration may be independent of circadian regulation. To test this hypothesis, we generated arrhythmic larvae by raising them immediately at the onset of fertilization under constant darkness (DD), wherein all key circadian clock genes lost their rhythmic expression (Fig. 5). Intriguingly, under this treatment, neutrophils migration is still reduced in these arrhythmic *aanat2* ^−/−^ larvae from day to night (Fig. 5), suggesting that endogenous melatonin directly regulates neutrophils migration, rather than through modulation of the circadian clock.

Cytokines have been shown to be rhythmically expressed^8, 45^ and exogenous melatonin can modulate their expression^42, 44, 46^. In this study, we found that both *il-1β* and *il-8* are significantly down-regulated in *aanat2* ^−/−^ larvae (Fig. 6), both of which have been shown to be able to attract neutrophils migration in zebrafish^4, 47^. These results implied that the promoting effect of endogenous melatonin on neutrophils migration may be mediated, at least in part, by cytokine signaling, although this hypothesis should be investigated in detail in the future.

Melatonin is known to play roles in the circadian clock and peripheral immune system^39, 48^. While a large number of studies have employed nocturnal animals such as mice or rats to investigate effects of melatonin on immune functions, only a few have used a diurnal model, such as zebrafish, to explore roles of endogenous melatonin on immune functions. Here we showed that endogenous melatonin modulates rhythmic neutrophils migration in diurnal zebrafish. Reduced melatonin levels often occur in the elderly and related patients^49^, implying that reduced endogenous melatonin may contribute partially to the subdued immune functions in the elderly and related patients. A deep understanding of how endogenous melatonin interacts with the immune system and other physiological functions using the *aanat2* ^−/−^ zebrafish may set the stage for developing novel therapies for the elderly and related patients.

## Electronic supplementary material

Endogenous melatonin promotes rhythmic recruitment of neutrophils toward an injury in zebrafish

## References

[1] Wang XG (2014). Inhibitors of neutrophil recruitment identified using transgenic zebrafish to screen a natural product library. Dis Model Mech.

[2] Renshaw SA (2006). A transgenic zebrafish model of neutrophilic inflammation. Blood.

[3] Henry KM, Loynes CA, Whyte MK, Renshaw SA (2013). Zebrafish as a model for the study of neutrophil biology. Journal of leukocyte biology.

[4] de Oliveira S (2013). Cxcl8 (IL-8) mediates neutrophil recruitment and behavior in the zebrafish inflammatory response. J Immunol.

[5] Deng Q, Harvie EA, Huttenlocher A (2012). Distinct signalling mechanisms mediate neutrophil attraction to bacterial infection and tissue injury. Cellular microbiology.

[6] Mathias JR (2006). Resolution of inflammation by retrograde chemotaxis of neutrophils in transgenic zebrafish. Journal of leukocyte biology.

[7] Niethammer P, Grabher C, Look AT, Mitchison TJ (2009). A tissue-scale gradient of hydrogen peroxide mediates rapid wound detection in zebrafish. Nature.

[8] Curtis AM, Bellet MM, Sassone-Corsi P, O’Neill LA (2014). Circadian clock proteins and immunity. Immunity.

[9] Boivin DB (2003). Circadian clock genes oscillate in human peripheral blood mononuclear cells. Blood.

[10] Arjona A, Sarkar DK (2005). Circadian oscillations of clock genes, cytolytic factors, and cytokines in rat NK cells. J Immunol.

[11] Keller M (2009). A circadian clock in macrophages controls inflammatory immune responses. Proceedings of the National Academy of Sciences of the United States of America.

[12] Shackelf PG, Feigin RD (1973). Periodicity of Susceptibility to Pneumococcal Infection - Influence of Light and Adrenocortical Secretions. Science.

[13] Feigin, R. D., Sanjoaqu, V. H., Haymond, M. W. & Wyatt, R. G. Daily Periodicity of Susceptibility of Mice to Pneumococcal Infection. Nature **224**, 379–&, doi:10.1038/224379a0 (1969). 10.1038/224379a0 5343888

[14] Gupta A, Shetty H (2005). Circadian variation in stroke - a prospective hospital-based study. International journal of clinical practice.

[15] Muller JE (1985). Circadian variation in the frequency of onset of acute myocardial infarction. The New England journal of medicine.

[16] Suarez-Barrientos A (2011). Circadian variations of infarct size in acute myocardial infarction. Heart.

[17] Ren DL, Li YJ, Hu BB, Wang H, Hu B (2015). Melatonin regulates the rhythmic migration of neutrophils in live zebrafish. Journal of pineal research.

[18] de Borsetti NH (2011). Light and melatonin schedule neuronal differentiation in the habenular nuclei. Developmental biology.

[19] Cazamea-Catalan D (2012). Functional diversity of Teleost arylalkylamine N-acetyltransferase-2: is the timezyme evolution driven by habitat temperature?. Molecular ecology.

[20] Klein DC (2007). A N-acetyltransferase: “the timezyme”. Journal of Biological Chemistry.

[21] Pena C, Rincon J, Pedreanez A, Viera N, Mosquera J (2007). Chemotactic effect of melatonin on leukocytes. Journal of pineal research.

[22] Ren DL (2015). Exogenous melatonin inhibits neutrophil migration through suppression of ERK activation. Journal of Endocrinology.

[23] Martins E (2001). Melatonin modulates allergic lung inflammation. Journal of pineal research.

[24] Carrillo-Vico A, Guerrero JM, Lardone PJ, Reiter RJ (2005). A review of the multiple actions of melatonin on the immune system. Endocrine.

[25] Laliena A (2012). Melatonin attenuates inflammation and promotes regeneration in rabbits with fulminant hepatitis of viral origin. Journal of pineal research.

[26] Beskonakli E (2000). The effect of pinealectomy on immune parameters in different age groups in rats: results of the weekly alteration of the zinc level and the effect of melatonin administration on wound healing. J Clin Neurosci.

[27] Molinero P, Soutto M, Benot S, Hmadcha A, Guerrero JM (2000). Melatonin is responsible for the nocturnal increase observed in serum and thymus of thymosin alpha1 and thymulin concentrations: observations in rats and humans. Journal of neuroimmunology.

[28] Delgobbo V, Libri V, Villani N, Calio R, Nistico G (1989). Pinealectomy Inhibits Interleukin-2 Production and Natural-Killer Activity in Mice. Int J Immunopharmaco.

[29] Cahill GM (2002). Clock mechanisms in zebrafish. Cell and tissue research.

[30] Hwang WY (2013). Efficient genome editing in zebrafish using a CRISPR-Cas system. Nature biotechnology.

[31] Jao LE, Wente SR, Chen WB (2013). Efficient multiplex biallelic zebrafish genome editing using a CRISPR nuclease system. Proceedings of the National Academy of Sciences of the United States of America.

[32] VanGuilder HD, Vrana KE, Freeman WM (2008). Twenty-five years of quantitative PCR for gene expression analysis. BioTechniques.

[33] Davie A, Sanchez JA, Vera LM, Sanchez-Vazquez J, Migaud H (2011). Ontogeny of the circadian system during embryogenesis in rainbow trout (Oncorhynchus mykyss) and the effect of prolonged exposure to continuous illumination on daily rhythms of per1, clock, and aanat2 expression. Chronobiology international.

[34] Falcon J (2001). Regulation of arylalkylamine N-acetyltransferase-2 (AANAT2, EC 2.3.1.87) in the fish pineal organ: evidence for a role of proteasomal proteolysis. Endocrinology.

[35] Tell RM, Kimura K, Palic D (2012). Rac2 expression and its role in neutrophil functions of zebrafish (Danio rerio). Fish & shellfish immunology.

[36] Nagy AD (2015). Melatonin adjusts the expression pattern of clock genes in the suprachiasmatic nucleus and induces antidepressant-like effect in a mouse model of seasonal affective disorder. Chronobiology international.

[37] Vriend J, Reiter RJ (2015). Melatonin feedback on clock genes: a theory involving the proteasome. Journal of pineal research.

[38] Torres-Farfan C (2011). A circadian clock entrained by melatonin is ticking in the rat fetal adrenal. Endocrinology.

[39] Stehle JH, von Gall C, Korf HW (2003). Melatonin: a clock-output, a clock-input. Journal of neuroendocrinology.

[40] Gandhi AV, Mosser EA, Oikonomou G, Prober DA (2015). Melatonin Is Required for the Circadian Regulation of Sleep. Neuron.

[41] Borges Lda S (2015). Melatonin decreases muscular oxidative stress and inflammation induced by strenuous exercise and stimulates growth factor synthesis. Journal of pineal research.

[42] Hung MW (2013). Melatonin ameliorates endothelial dysfunction, vascular inflammation, and systemic hypertension in rats with chronic intermittent hypoxia. Journal of pineal research.

[43] Barlas A (2004). Melatonin protects against pancreaticobiliary inflammation and associated remote organ injury in rats: role of neutrophils. Journal of pineal research.

[44] Lopes C, deLyra JL, Markus RP, Mariano M (1997). Circadian rhythm in experimental granulomatous inflammation is modulated by melatonin. Journal of pineal research.

[45] Hrushesky WJ, Langevin T, Kim YJ, Wood PA (1994). Circadian dynamics of tumor necrosis factor alpha (cachectin) lethality. J Exp Med.

[46] Lee YD (2009). Melatonin attenuates lipopolysaccharide-induced acute lung inflammation in sleep-deprived mice. Journal of pineal research.

[47] Yan B (2014). IL-1beta and reactive oxygen species differentially regulate neutrophil directional migration and Basal random motility in a zebrafish injury-induced inflammation model. J Immunol.

[48] Reiter RJ (1993). The melatonin rhythm: both a clock and a calendar. Experientia.

[49] Hardeland R, Madrid JA, Tan DX, Reiter RJ (2012). Melatonin, the circadian multioscillator system and health: the need for detailed analyses of peripheral melatonin signaling. Journal of pineal research.

