# Supplementary material for: Endogenous melatonin promotes rhythmic recruitment of neutrophils toward an injury in zebrafish
Source: Sci Rep. 2017 Jul 5;7:4696. doi: 10.1038/s41598-017-05074-w (PMC5498597; doi:10.1038/s41598-017-05074-w)
Supplement: Supplementary file 1 — Endogenous melatonin promotes rhythmic recruitment of neutrophils toward an injury in zebrafish [file 41598_2017_5074_MOESM1_ESM.pdf]

Endogenous melatonin promotes rhythmic recruitment of neutrophils toward an injury in zebrafish

Da-long Ren[1#*], Cheng Ji[2,3#], Xiao-Bo Wang[1], Han Wang[2,3*], Bing Hu[1*]

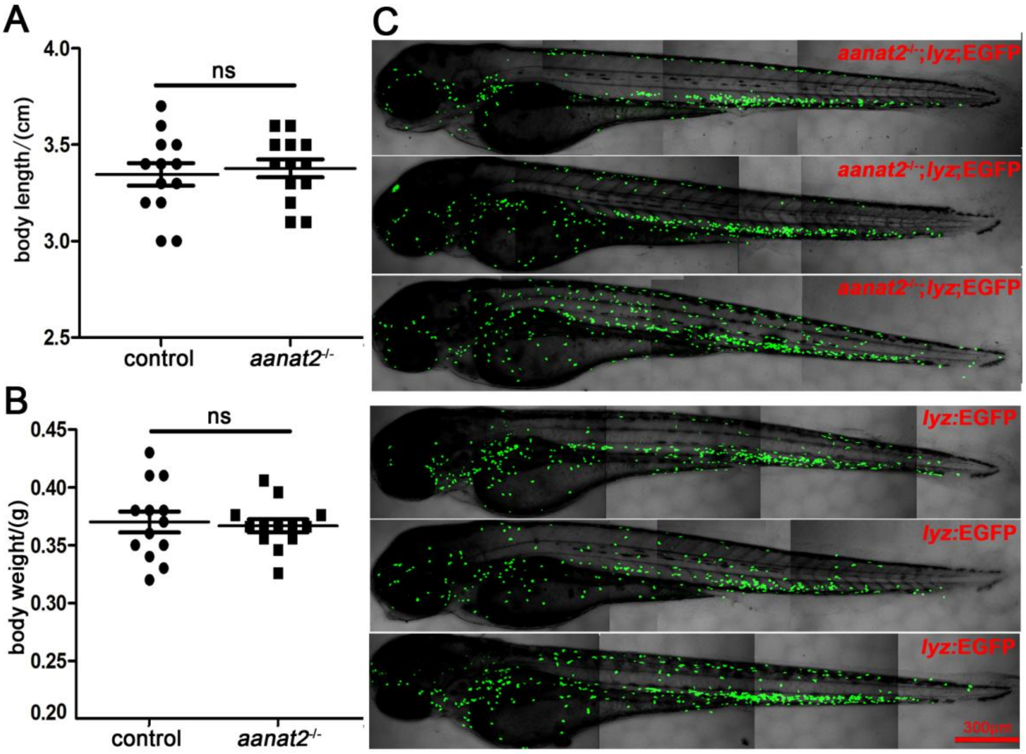

Supplementary Figure S1. No significant changes of body length, body weight and neutrophil distribution in *aanat2* mutant zebrafish.

(A, B) Body length and body weight of adult zebrafish (4 months years old) were not significantly different between the wild type and *aanat2* mutant groups (n=13, unpaired Student's *t*-test). **(C)** Confocal imaging of the whole larvae (4 days post fertilization) showed that the *aanat2* mutation did not cause a change in the visualized neutrophil distribution and number.

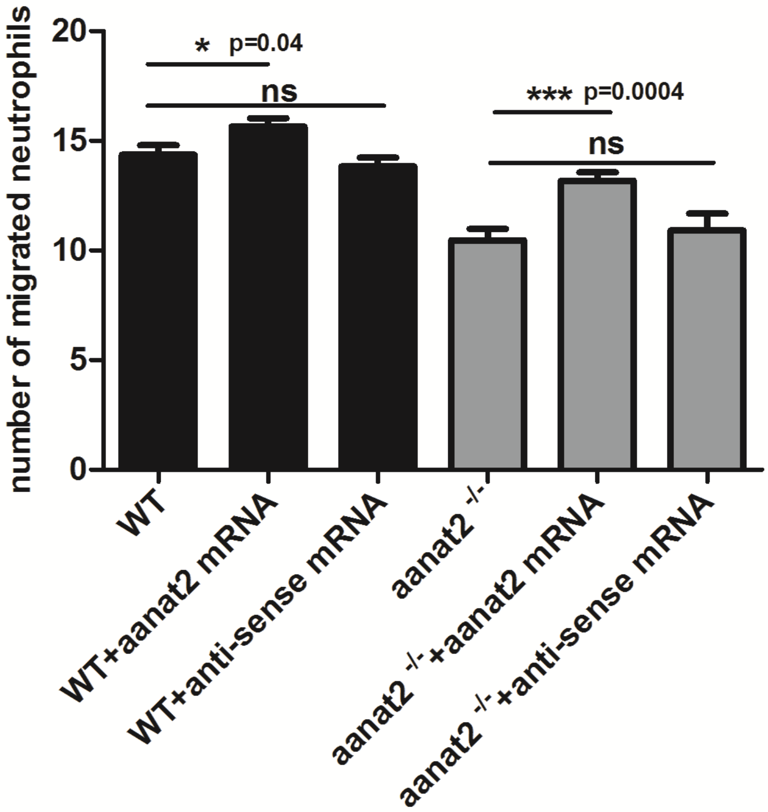

Supplementary Figure S2. Rescue of neutrophil migration by wild-type *aanat2* mRNAs and anti-sense mRNA.

200 ng/μl *a*anat2 capped mRNAs and anti-sense capped mRNAs were microinjected into one-cell of zebrafish *lyz*:EGFP embryos and *lyz:*EGFP;*aanat2*$^{-/-}$ embryos. None microinjected embryos of *lyz*:EGFP or *lyz:*EGFP;*aanat2*$^{-/-}$ were controls. The injury was conducted at 12:00 in the day. The data showed that *aanat2* mRNA can partly rescued the neutrophils migtaion and the anti-sense mRNA had no significant effect (every group, n=40). (*P<0.05, ***P<0.001).

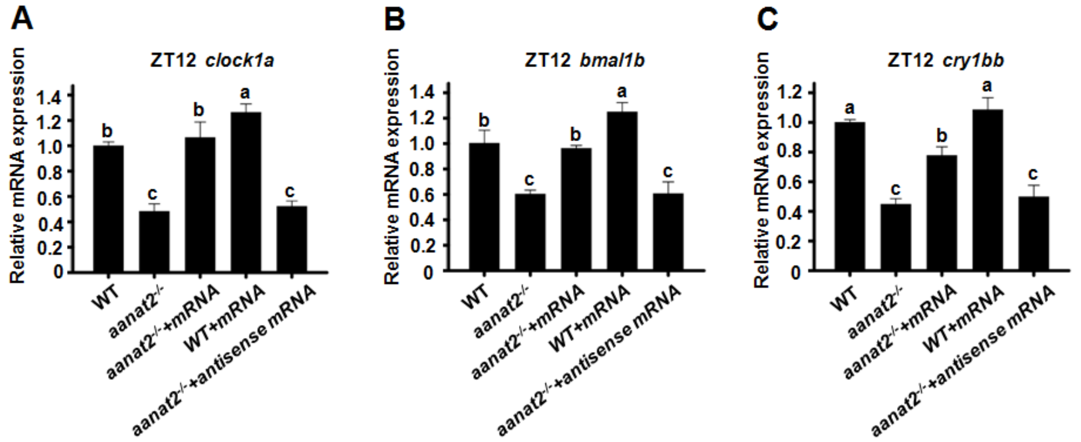

Supplementary Figure S3. Rescue of clock gene expression by wild-type *aanat2* and anti-sense mRNAs.

200 ng/μl *aanat2* capped mRNAs or 200 ng/μl anti-sense capped mRNAs were microinjected into one-cell of zebrafish wild type embryos or *aanat2*$^{-/-}$ embryos. None microinjected embryos of wild type or *aanat2*$^{-/-}$ were as controls. Total RNAs were extracted from 50 larvae at ZT12 each sample. The data was analyzed from three samples. **(A-C)** qRT-PCR analysis showed relative mRNA expression of *clock1a*, *bmal1b* and *cry1bb*, respectively The genes were relative expression to β-actin (ANOVA analysis). Mean values with different letters are significantly different (*P*<0.05).

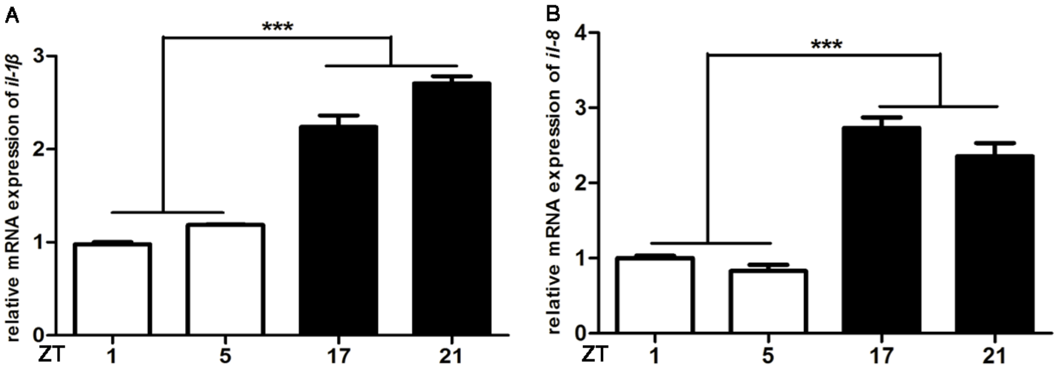

Supplementary Figure S4. *Il-1β* and *il-8* mRNA expression in day and night.

Total RNA was extracted from 50 embryos in day and night. Quantitative real-time PCR (qRT-PCR) was conducted with the SYBR green system. The clock and cytokine genes were amplified using the profiles of 95 ℃, 10 s, 60 ℃, 30 s for 40 cycles. qRT-PCR was performed in triplicate with three individual biological samples at corresponding time points, and the results were normalized to the expression level of the housekeeping gene β-actin and shown as a relative expression level calculated using the $2^{-\Delta\Delta Ct}$ method. *P* values were analyzed with one-way analysis of variance (ANOVA) test. ZT1 and ZT5: day time; ZT17 and ZT21: night time.
